# Vitamin C-induced oxalate nephropathy: a case report

**DOI:** 10.3402/jchimp.v2i2.17718

**Published:** 2012-07-16

**Authors:** Harmeet Gurm, Mohamed Ali Sheta, Noel Nivera, Allan Tunkel

**Affiliations:** 1 Department of Internal Medicine, Monmouth Medical Center, Long Branch, NJ, USA; 2 Department of Internal Medicine, Section of Nephrology, Monmouth Medical Center, Long Branch, NJ, USA

**Keywords:** vitamin C, oxalate nephropathy, acute kidney injury

## Abstract

Therapeutic benefits of vitamin C is an area of active research and large doses have been suggested by many studies for treatment of various conditions. We are describing a case of oxalate nephropathy leading to end stage kidney disease, which occurred secondary to mega-dose of oral vitamin C. Increasing the awareness between medical personnel as well as patients will clearly decrease the incidence of this debilitating but, at the same time, highly preventable disease.

Vitamin C is an essential water-soluble vitamin. Dietary reference intake (DRI) for vitamin C is 75 mg/day for most adults. Up to 2 g/day can be tolerated orally (1). However, larger doses of vitamin C have been utilized for treatment of various conditions. A recent study recommended 84 mg/day of oral vitamin C to reduce symptoms, duration, and frequency of the common cold (2). In a meta-analysis, a 500 mg daily dose was found to be effective in lowering the serum concentrations of low-density lipoprotein cholesterol and triglycerides (3). Choi et al. (4) prospectively studied the relation between vitamin C and incidence of gout over 20 years, and found that higher vitamin C intake was independently associated with a lower risk of gout. In addition, several other studies suggested a role for vitamin C in both prophylaxis and treatment of some malignancies. Unfortunately, ingestion of large doses of vitamin C may be associated with adverse events. Here we describe a case of oxalate nephropathy secondary to ingestion of excessive doses of oral vitamin C.

## Case report

A 58-year-old woman with a history of stable celiac disease since infancy presented with a 5-week history of generalized weakness, reduced appetite and halitosis described as an ‘ammonia-like’ smell. She had no urinary complaints. Blood work revealed a BUN of 120 mg/dl and creatinine of 9.1 mg/dl; routine blood work, including tests of renal function, done 3 months earlier was normal. On physical examination, her vital signs were: Temperature 98.9° Fahrenheit, Radial pulse of 70/min, BP in right arm in supine position was 123/76 mmHg, respiratory rate of 18 breaths/min and SpO2 of 100% on room air. She had no orthostatic hypotension, and the remainder of the physical examination was normal. An arterial blood gas taken while the patient breathed ambient air showed a pH of 7.45, pCO_2_ 28 mmHg, pO_2_ 113 mmHg, HCO_3_ 20 mmol/L. Her serum chemistries revealed sodium of 138 mEq/L, potassium of 3.9 mEq/L, chloride of 95 mEq/L, and bicarbonate of 24 mEq/L. Her urinalysis revealed trace protein and 6–10 RBCs per high power field, WBCs 0–5 per high power field, but was positive for eosinophils. Her urine culture did not show any significant bacteriuria. The calculated fractional excretion of sodium was 3.51, with a serum and urine anion gap of 19 and 35, respectively.

**Fig. 1:**
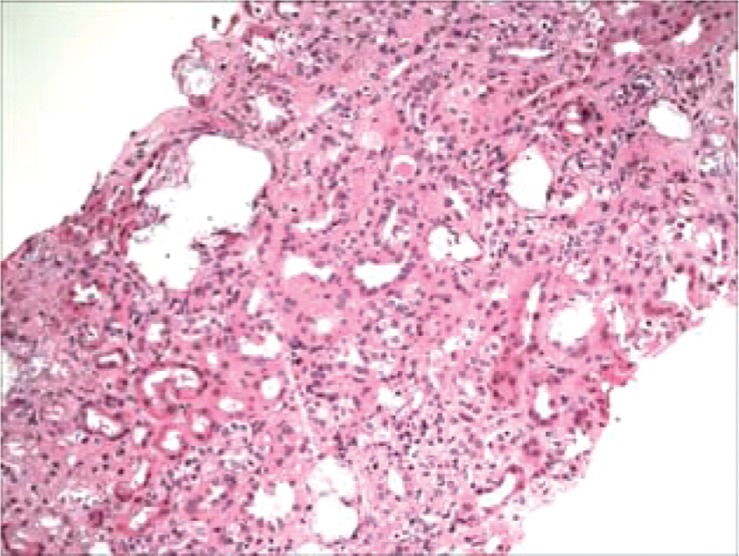
H&E Slide showing multiple tubular distention by a clear crystalline material with mild to moderate mononuclear inflammation.

**Fig. 2:**
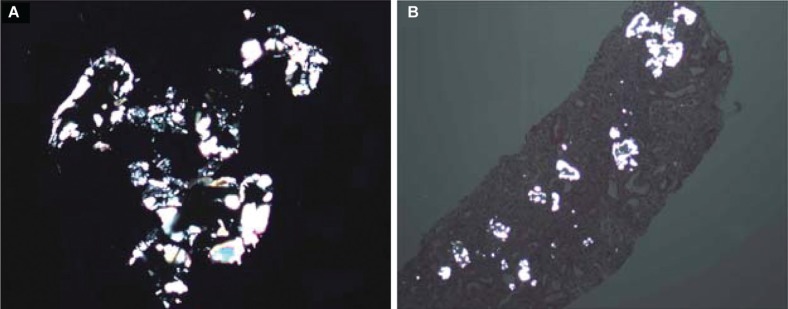
Polarized oxalate in tubules (A and B).

The patient did not show any significant response to supportive measures and therefore underwent hemodialysis. A renal ultrasound showed increased renal parenchyma echogenicity; kidney size was adequate bilaterally (right kidney: 9.6×4.4×5.8 cm and left kidney: 11.7×5.1×6.2 cm). Serum anti-glomerular basement membrane antibody, cANCA, and pANCA were negative. Anti-double stranded DNA and hepatitis serologies were negative, and serum complement concentrations were normal. She was also negative for any evidence of secretory myeloma. The patient underwent a kidney biopsy, which revealed severe oxalate nephropathy, with tubular atrophy and interstitial fibrosis (Figs. 1 and 2A, B). The electron microscopy also confirmed the tubular injury with oxalate crystals.

Upon further questioning, the patient denied use of any ethylene glycol products. However, she admitted the use of a highly concentrated powder of vitamin C, which contained 814 mg of vitamin C and 100 mg of calcium ascorbate in quarter of a teaspoon. She was taking one to two teaspoons of this highly concentrated powder daily for approximately 1 month, accounting for an approximate daily consumption of 3–6.5 g of vitamin C. After almost 1 year of follow-up and no further ingestion of vitamin C, she remains hemodialysis-dependent and is being currently evaluated for renal transplantation.

## Discussion

Oxalate nephropathy is characterized by tubular crystalline deposits of calcium oxalate leading to acute and chronic tubular injury, interstitial fibrosis, and progressive renal insufficiency. In one study, oxalate deposition was recognized in 1% of native kidney biopsies (5). In another review of 100 renal biopsies performed at Columbia University, oxalate nephropathy was found to be the etiology of acute kidney injury in 2.1% of elderly patients (6). Hyperoxaluria is either primary or secondary. There are three recognized types of primary hyperoxaluria (PH): type 1 is due to defects in the gene that encodes the hepatic peroxisomal enzyme alanine glyoxylate aminotransferase (AGT); type 2 is due to defects in the gene that encodes the cytosolic enzyme glyoxylate reductase/hydroxypyruvate reductase (GRHPR); and type 3 is due to mutations in the uncharacterized gene DHDPSL. Secondary hyperoxaluria results from increased absorption in patients with intestinal malabsorption syndromes, direct dietary consumption of oxalate rich products, or ingestion of other substances (e.g., vitamin C), which may be metabolized into oxalate (7).

Vitamin C (ascorbic acid, ascorbate) is an essential micronutrient involved in many biologic and biochemical functions. Unlike plants and most of animals, humans cannot synthesize vitamin C because they lack the enzyme I-gulonolactone oxidase. Oral bioavailability in doses up to 200 mg is 100%, but declines markedly at intakes greater than 1 g (8). The reduced form of vitamin C is absorbed in the upper ileum by an active sodium-dependent process involving specific vitamin C transporters (9). However, the oxidized form of the vitamin is transported by facilitated-diffusion glucose transporters, GLUT 1, 3 and 4 (10). In the renal glomeruli, vitamin C is filtrated freely and actively reabsorbed by the renal tubules, and the factor, which limits this reabsorptive process, is the existence of a maximal rate (11). Also, vitamin C is catabolized to oxalic acid, which is urinary excreted.

The multiple potential benefits, lack of studies discussing the possible adverse effects of large doses, and the availability of vitamin C over the counter are the main reasons behind the inappropriate use of vitamin C. In a survey done during the annual meeting of the complementary and alternate medicine in 2006, 172 practitioners administered intravenous vitamin C to 11,233 patients; the average dose was 28 g every 4 days, with 22 total treatments per patient (12). On the other hand, several case reports described oxalate nephropathy complicated by kidney injury in much lower doses than the tolerable upper intake level (2 g/day) (13–15). As a result of our observation, cautious use of vitamin C should be considered irrespective of baseline renal function.

## Conclusion

In spite the multiple potential benefits of vitamin C, we strongly recommend cautious use of large doses of vitamin C irrespective of baseline renal status. We believe that oxalate nephropathy secondary to vitamin C can be prevented by increasing the awareness between physicians as well as patients.
